# The Argentinian mother-and-child contaminant study: a cross-sectional study among delivering women in the cities of Ushuaia and Salta

**DOI:** 10.1080/22423982.2017.1364598

**Published:** 2017-08-28

**Authors:** Inger Økland, Jon Øyvind Odland, Silvinia Matiocevich, Marisa Viviana Alvarez, Torbjørn Aarsland, Evert Nieboer, Solrunn Hansen

**Affiliations:** ^a^ Department of Obstetrics and Gynecology, Stavanger University Hospital, Stavanger, Norway; ^b^ Department of Community Medicine, Faculty of Health Sciences, UiT The Arctic University of Norway, Tromsø, Norway; ^c^ Banco de Sangre, Clínica San Jorge, Ushuaia, Argentina; ^d^ Pediatric Department, Hospital Público Materno Infantil, Salta, Argentina; ^e^ Department of Research, Stavanger University Hospital, Stavanger, Norway; ^f^ Department of Biochemistry and Biomedical Sciences, McMaster University, Hamilton, Ontario, Canada

**Keywords:** Pregnancy, persistent toxic substances (PTS), human levels, Argentina

## Abstract

Several ongoing international multidisciplinary projects have examined linkages between environmental chemicals and health. In contrast to Arctic regions, information for the Southern Hemisphere is scarce. Because of the inherent practice of pesticide utilisation and mismanagement, food security is potentially threatened. The most vulnerable period in human life occurs during pregnancy and early childhood, thus a focus on the body burdens of PTS in pregnant or delivering women is warranted. The current study was designed to investigate health risks related to exposure to PTS and food security in two regions of Argentina (Ushuaia and Salta). Our aims were to quantify concentrations of organic and inorganic toxins in serum or whole blood of delivering women and to collect pertinent dietary and medical information. The overall study design, the basic demographic features and essential clinical chemistry findings are described in the current paper. The socioeconomic differences between the two study areas were evident. On average, the women in Ushuaia were 4 years older than those in Salta (28.8 vs. 24.7 years). Respectively, the proportion of current smokers was 4.5 vs. 9.6%; and Salta had a higher birth rate, with 15.6% being para four or more. Saltanean women reported longer breastfeeding periods. Caesarean sections were more frequent in Ushuaia, with 43% of Caesarean deliveries compared with only 6% in Salta. Employment was high in both communities. Recognised environmental pollution sources in the vicinity of participant dwellings were widespread in Salta (56.1%) compared to Ushuaia (9%). The use of pesticides for insect control in homes was most common in Salta (80%). There is an urgent need for a comprehensive assessment of exposures in areas of the Southern Hemisphere. Our data set and the planned publications of observed concentrations of inorganic and organic environmental contaminants in both mothers and their newborns will contribute to this objective.

## Introduction

A number of persistent toxic substances (PTS), such as persistent organic pollutants (POPs) and certain toxic metals (especially cadmium, lead and mercury), are recognised as being responsible for adverse development and health effects in children [1–5]. Foetuses and newborns are particularly sensitive to the toxic effects of certain metals and organochlorine environmental pollutants that are resistant to degradation – they have long environmental life times (often in years). Due to their bioaccumulation along the food chain, the diet has become the primary source for humans. Foetal exposure occurs via the umbilical cord, while for breast-fed infants mother’s milk can constitute a primary source. Clearly, maternal exposure is of special relevance [6–8]. The levels of PTS in maternal blood during pregnancy usually constitute a measure of the potential foetal risk. Of particular concern are long-term negative impacts on a child’s mental development, immunity to disease, and future reproductive health and cancer risk.

Several ongoing multidisciplinary study projects assess human exposure to PTS in different geographical regions of the world to establish linkages between environmental chemicals and health. In general, the coastal populations of the Arctic are known to have high body burdens, especially those consuming marine fish and mammals [9]. The Arctic Monitoring and Assessment Programme (AMAP) started in 1991 and currently all Arctic countries are members, namely: Canada, Denmark, Finland, Iceland, Norway, Russia, Sweden and USA [9–12]. Countries located in tropical and southern areas have joined more recently, including South Africa [8,13], Brazil [6,14], Vietnam [15] and Australia [16,17]. The extent of human exposure, as measured by biological contaminant concentrations, has not been adequately investigated in South America. As Argentina features the most southern towns and settlements in the world, it is relevant to compare exposures in the Southern-Hemisphere Antarctic with those known for the northern Arctic. Furthermore, Argentina also allows a comparison between high altitude populations with coastal inhabitants.

Due to the agricultural profile of South American countries and the inherent practice of pesticide utilisation, mismanagement of the latter constitutes an important environmental problem for this continent [18]. Furthermore, widespread land degradation and deforestation have taken place in productive areas as a consequence of poor governance and lack of plans for using natural resources [19]. Such activities inevitably accelerates climate change and, thus, land degradation accompanied by increased water pollution have negative consequences for biodiversity, the natural environment and human health. These changes have high economic costs that lead to increased poverty. The rising temperature related to the climate change will also alter human exposure to environmental contaminants significantly, especially for metals released from icebergs, oceans and rivers (including mercury) [20]. Furthermore, extensive distribution by long-range transport across oceans implies that remote areas (regarded initially as environmentally clean) receive contaminants that are transferred to the food web and humans [9]. Due to the volatility and particulate properties of contaminants and the dominant atmospheric and oceanic south–north circulations, contaminants have been shown to be transported towards the circumpolar Arctic regions of the Northern Hemisphere [9]. In comparison, there is less knowledge about comparable long-distance transfer in the Southern Hemisphere.

In the current paper we describe the framework for an AMAP-compatible study that was given the acronym EMASAR (*Estudio del Medio Ambiente y la Salud Reproductiva* – Study on Environment and Reproductive Health). It was designed to investigate maternal/foetal health risks related to food security and exposure to PTS in two regions of Argentina and food. The EMASAR assessed concentrations of environmental toxins in the blood of delivering women and collected pertinent personal, dietary and medical information. More specifically, we describe the overall study design and report and compare basic demographic features of the two study populations and clinical chemistry findings for selected residents of the Argentinian cities of Salta in the northwest and Ushuaia in the south. The current article constitutes the background and context for a number of biomonitoring publications concerning PTS exposures.

## Materials and methods

### Geographical information

**Figure 1.**
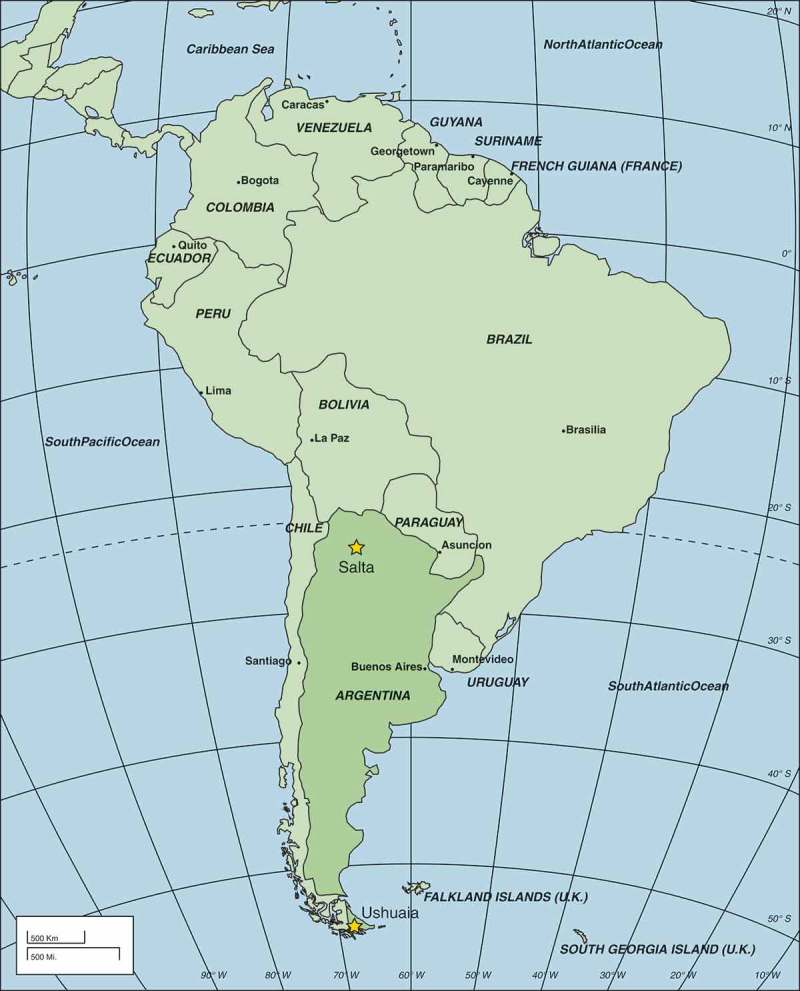
Map of South America with the study areas Salta and Ushuaia, Argentina.

The geographical locations of the EMASAR study sites are shown in Figure 1. The City of Salta is the capital of Salta Province, which is located in the Lerma Valley of the Andes Mountains foothills and is at 24.5° south of the Equator. The metropolitan area has a population of around 620,000 inhabitants, which makes it the second most populated city in northwestern Argentina, while it is 1,210,000 for Salta Province. Its climate is highland subtropical, with average monthly temperatures ranging from 12 to 22°C. Based on its inland location and its livestock economy and tradition, beef is the main component of the diet. Salta’s economy is diverse, but relatively under-developed; poverty is a general feature and there are large socioeconomic inequalities.

Ushuaia is the capital of the Argentinian Province of Tierra del Fuego, Antártida e Islas del Atlántico Sur. It is the southernmost city in the world, located at sea level and 54.5° south of the Equator and has a sub-polar oceanic climate. The average monthly temperature varies from 1 to 10°C. The city has a population of some 60,000 people, while the province has around 130,000 inhabitants. The main economic activities in Ushuaia are fishing, natural gas and oil extraction, sheep farming and ecotourism. Having a free port status, the city also attracts advanced industry with salaries above the national standards. Qualified personnel come from other parts of Argentina to work for both short and long periods. Consequently, the population of Ushuaia is neither homogenous nor stable over time, while the socioeconomic conditions are among the most prosperous in Argentina.

### Study design and study populations

EMASAR is an observational study, with a cross-sectional design that was compatible with the circumpolar programme in the context of the AMAP [9]. The field work was conducted at the Hospital Público Materno Infantil de Salta and at the Clínica San Jorge in Ushuaia. The hospital in the City of Salta is a public institution, which is responsible for all in-hospital deliveries in the city and is the referral hospital for the province. Clínica San Jorge in the City of Ushuaia is a private institution co-responsible with a public hospital for the in-hospital deliveries in the city and the surrounding provincial areas.

EMASAR is a collaborative project between the UiT The Arctic University of Norway, Tromsø, the Stavanger University Hospital, Stavanger (Norway) and the two Argentinian partner hospitals mentioned. During the preparation period and throughout the study period, the project group carried out several organisational meetings at the two study sites. The local research co-workers were thoroughly informed about the protocol and the sampling procedures. All the questionnaires were prepared in English and subsequently translated into Spanish. Individual pre-numbered sampling kits were assembled in Norway and included printed questionnaires, blood sampling equipment, sample transfer vials and comprehensive written instructions for the local staff.

Recruiting of the study cohort took place over the period April 2011–March 2012 and included women who either were about to deliver or had given birth within the last 48 hours at one of the two hospitals. The participants were recruited shortly before or at admission to the delivery unit. The mothers had to be above 18 years of age, be willing to participate and written informed consent was required. We estimate that more than 90% of invited women consented to participate, although refusals were not systematically registered. A total of 717 women were recruited; of these, 19 from Salta were excluded due to a lack of biological samples, yielding a final study population of 698 (200 from Ushuaia and 498 from Salta).

The study (#2010/7317) was approved by the Ethics Committee of the Salta Medical Association and the Ministries of Health in both provinces. As required by law, the Norwegian Regional Committee for Medical and Health Research Ethics (REC North) approved the study (#2011/706). The study was conducted in accordance with the Helsinki declaration.

### Data collection and analyses

#### Questionnaires

Participants were examined and interviewed by the presiding midwife or obstetrician. The questionnaire was based on that used in comparable studies [13,15], with additional questions and adjustments pertaining to the Argentinian context. The information collected included: maternal age, previous children and breastfeeding experiences, medical history (e.g. chronic diseases, number of amalgam fillings and silicone implants), socioeconomic factors (place of birth, family conditions, housing, education, employment), environmental factors (use of fuel for warming or cooking, potential exposures to chemicals/pollution/pesticides at work or at home), lifestyle (use of coffee, tea/maté, tobacco, coca leaves and alcohol) and information on diet before and during pregnancy.

The questions about diet pertained to the frequency of intake (never or seldom, at least once a week or almost every day) of various basic food categories: eggs, meat (red meat, poultry, processed or tinned meat); fish (tinned, smoked, processed, freshwater or seafood); fruits and vegetables (root, leafy/ground and other); dairy products (milk, butter, cheese); fats (oils, margarine); carbohydrates (cereals, noodles, pasta, bread, sugar); and fluids (juices, soft drinks, bottled water). We also inquired about the use of vitamin supplements and personal care products (e.g. creams, lotions, sun protection products, deodorant, soap, hair products/hair dye and cosmetics).

#### Clinical information

Clinical obstetrical data are based on hospital records and a medical doctor completed a standardised form. Information on the history of earlier pregnancies was also sought, along with obstetrical and neonatal data for the current delivery. The latter included date of delivery, gestational age (as clinically estimated; Naegele term), as well as the weight, length, head circumference, sex of the neonate and any notable malformation.

#### Sampling and analyses

Maternal blood, urine and hair samples were obtained at 36±12 hours after the delivery (optimally in the morning). Use of personal products prior to blood sampling, time since last meal and last cup of coffee, as well as time of blood sampling and of freezing were recorded. Measurement of maternal height (in cm) and weight (in kg) were obtained after the delivery and, when possible, coincided with the blood sampling.

Non-fasting venous blood samples were collected from the antecubital vein from the mothers. For the chemical analyses of toxic and essential elements, blood was drawn into a BD Vacutainer® for trace elements (BD Hemogard™ Royal Blue, Ref# 368381; plastic, 6-ml, with 10.8 mg K_2_ EDTA) and transferred into pre-rinsed cryo-vials (Sarstedt CryoPure 5.0 ml and 2.0 ml tube). Additionally, five BD Vacutainers® (BD SST II Plus Advance 10/8.5 ml) were sampled after centrifugation at 2000 relative centrifugal force (RCF) for 10 minutes. The maternal serum was then transferred into four cryo-vials (Sarstedt CryoPure 2.0 ml tubes) for general analyses, was subsequently apportioned into three glass vials (4.0 ml, pre-rinsed with *n*-hexane/acetone) and retained for chemical analyses of POPs and selected haematological/hormonal parameters. The latter were analysed immediately at the local hospital laboratories in Salta and Ushuaia and included the routine analyses of reactive protein C (CRP), ferritin, follicle-stimulating hormone (FSH), luteinizing hormone (LH), prolactin, oestrogen, progesterone and lipid profile (cholesterol, triglycerides, phospholipids, HDL, LDL).

Blood samples from the newborns were collected as part of routine sampling at the hospital. Venous samples were drawn from the heel capillary with standard equipment into vials (BD Microtainer® Brand Tubes, 2.0 ml). Samples were centrifuged at 1200 RCF for 10 minutes and the plasma was transferred into cryovials (Sarstedt CryoPure 2.0 ml tubes).

A maternal morning spot urine was also collected (Sarstedt inc., Newton, NC), which was transferred into two 10 ml vials (BD Falcon; Becton Dickinson (BD), Plymouth, UK). Maternal hair strands (~5 cm length) were taken close to the scalp from the occipital lobe and tied with a cotton thread and subsequently stored in an envelope at room temperature until shipping to Norway.

Both the urine and blood samples collected for contaminant analyses were frozen immediately after preparation and stored at −20°C at the local hospital until they were shipped frozen to Norway and stored in the EMASAR biobank at the the UiT The Arctic University of Norway at −35°C until analysis. For the chemical analyses, blood samples were later transferred in a frozen state to the Department of Environmental Chemistry, Institute of Environmental Assessment and Water Research (IDAEA), Spanish Council for Scientific Research, Barcelona, Spain for chemical analyses of persistent organic pollutants and to the Department of Environmental Sciences, “Jožef Stefan” Institute (JSI), Ljubljana, Slovenia for determination of toxic and essential elements. More detailed descriptions of the analytical procedures and the quality control measures employed will be reported in upcoming articles.

### Follow-up of the children

The participating mother was invited to bring her child to a 1-year follow-up examination during which a clinical examination was performed and a questionnaire on the health and development of the child during the first year of life was completed. Anthropometric measurements were also made (specifically weight, height, head and abdominal circumference).

### Statistical analyses

Statistical analyses were carried out using the IBM SPSS-Statistics for Windows statistical package version 24 (SPSS Inc. Chicago, IL). For comparisions of information between the two study sites, non-parametric tests were performed for interval data with non-normal distributions; Kruskal Wallis or Mann–Whitney U-test when unequal or equal variance, respectively. For categorical data, the Mann–Whitney U-test was used. The level of significance was set at p < 0.05.

## Results

Some personal and obstetrical characteristics of the women and the pregnancy outcomes in the study are presented in Tables 1 and 2, respectively. The women in Ushuaia were, on average, 4 years older than the women in Salta (28.8 vs. 24.7 years). In Ushuaia, most women were married/cohabitants, while in Salta one third were single; the latter were less educated, had more amalgam fillings and the proportion of current smokers was higher (9.6 vs. 4.5%). Nevertheless, the overall smoking rate prior to pregnancy was comparable, but significant (≥ 25% did so). More Saltanean women were exposed to passive smoking at home (42 vs. 28%). The mean pre-pregnancy body mass indeces (BMIs) were the same, namely 24. During pregnancy, Ushuaian women gained 5 kg more body weight than those in Salta. The women in Ushuaia had in general more permanent work compared to the Salta women (66% vs. 17%). Selected maternal and newborn characteristics are summarised in Table 2. The proportion of first-time mothers was 44% in Salta and 41% in Ushuaia and the former had a higher birth rate, with 16% being para four or more. Saltanean women reported longer breastfeeding periods and fewer used vitamin and folic acids supplements both before and during pregnancy. According to the medical reports, gestational diabetes, hypertensive disorders and anaemia (Hgb < 9.0 g/dL) were almost non-existent among the women from Salta, with only one case of each reported; by contrast in Ushuaia there were 12, 11 and 2 cases, respectively (data not shown). The births took place at a gestational age close to 39 weeks at both sites, with a range of 32–41 for Ushuaia and 33–42 for Salta. Caesarean sections were more frequent in Ushuaia, specifically 43% of the deliveries compared to only 6% in Salta. The newborns in Ushuaia were slightly heavier, as shown in Table 2.

**Table 1.** Personal characteristics of the study population from Ushuaia and Salta.

	Ushuaia (n = 200)				Salta (n = 98)				
	n	Mean (SD) or n (%)	50th	min–max	n	Mean (SD) or n (%)	50th	min–max	p-value^a^
Age	200	28.8 (6.5)	28	16–45	498	24.7 (6.2)	23	14–44	***
≤20		28 (14.0)				177 (35.5)			
21–25		45 (22.5)				133 (26.7)			
26–30		55 (27.5)				102 (20.5)			
31–35		38 (19.0)				57 (11.4)			
>35		34 (17.0)				29 (5.8)			
Marital status	200				498				***
Married		72 (36.0)				56 (11.2)			
Cohabiting		106 (53.0)				283 (56.8)			
Divorced		1 (0,5)				3 (0.6)			
Single		21 (10.5)				156 (31.3)			
Education	200				498				***
Primary		7 (3.5)				161 (32.3)			
Secondary		97 (48.5)				284 (57.0)			
Tertiary		56 (28.0)				40 (8.0)			
University		40 (20.0)				12 (2.4)			
Permanent job	200	132 (66.0)			494	84 (17.0)			
Height, cm	200	162 (6.0)	162	147–181	496	158 (5.8)	158	140–176	***
Weight, pre-pregnancy, kilo^b^	192	62 (11.0)	60	40–111	449	59 (11.0)	57	35–109	***
BMI, pre-pregnancy, kg/m^2^	192	24 (4.0)	23	16–41	447	24 (4.2)	23	15–40	
Weight, post-partum, kilo	200	74 (11.0)	72	50–120	490	65 (11.2)	64	40–111	***
Smoking, current	200	9 (4.5)			498	48 (9.6)			*
Smoking last year	200	57 (28.5)			498	127 (25.5)			
Home indoor smoking	200	56 (28.0)			495	208 (42.0)			**
Amalgam fillings ever	199	89 (44.7)			481	338 (70.3)			***
Year of living current home	200	6.7 (7.6)	3	1–32	497	11.1 (9.7)	9	1–42	***
Nationality	200				497				
Argentina		197 (98.5)				482 (96.8)			
Other^c^		3 (1.5)				15 (3)			
Year of examination	200				498				***
2011		200 (100)				366 (73.5)			
2012						132 (26.5)			

^a^*, **, *** represent p-value <0.05, 0.010, 0.001, respectively; ^b^ based on self-reported pre-pregnancy weight; ^c^ Chile, Peru, Korean (Ushuaia); Bolivia (n = 13) and Paraguay (n = 2) (Salta).

**Table 2.** Selected maternal and newborn characeristics for the two study groups.

	Ushuaia (n = 200)				Salta (n = 498)				p-value^a^
	n	Mean (SD) or n (%)	50th	min-max	n	Mean (SD) or n (%)	50th	min-max	
Parity^b^	200	1.9 (0.96)	2	1–7	498	2.2 (1.5)	2	1–8	**
1		82 (41.0)				221 (44.4)			
2		75 (37.5)				121 (24.3)			
3		33 (16.5)				78 (15.7)			
≥4		10 (5.0)				78 (15.6)			
Previous breastfeeding, month	116	20.4 (19.6)	16	0–156	268	34.3 (29.0)	24	0–217	***
Vitamin supplements before pregnancy	198	30 (15.2)			493	32 (6.5)			***
Vitamin supplements during pregnancy	198	77 (38.9)			490	137 (28.0)			**
Folic acid before pregnancy	196	46 (23.5)			482	42 (8.7)			***
Folic acid during pregnancy	199	168 (84.4)			494	289 (58.5)			***
Caesarean Section, elective	199	46 (23.0)			498	9 (1.8)			***
Caesarean Section, acute	199	41 (20.5)			498	19 (3.8)			***
Gender newborn, girl	*200*	104 (52)			479	257 (53.7)			
Length, cm	198	49.6 (2.1)	50	42–54	492	48.5 (2.2)	49	41–55	***
Weight, kilo	198	3.38 (0.44)	3.38	2.12–4.50	491	3.29 (0.48)	3.3	1.65–5.20	*
Head, cm	197	34.9 (1.5)	35	31–40	491	34.3 (1.4)	34	28–38	***
Gestational age, weeks	199	38.8 (1.4)	39	32–41	461	38.8 (1.3)	38.8	33–42	

^a^*, **, *** represent *p*-value <0.05, 0.010, 0.001, respectively; ^b^ Total number of childbirths including stillborns after week 23. Twins count as one birth and one pair of twins is included.

Various characteristics concerning housing, family situation and social environment are summarised in Table 3. In both communities the participants lived in homes, although almost twice as many people lived together in the same house in Salta (seven vs. four in Ushuaia). In Salta, most (~80%) owned their home, while in Ushuaia more dwellings were rental units and there was more dwelling place mobility. Although partners in Salta had lower education, employment was high in both communities and most study participants resided in urban areas.

**Table 3.** Environmental characteristics of the study population from Ushuaia and Salta.

	Ushuaia (n = 200)				Salta (n = 498)				p-value^a^
	n	Mean (SD) or n (%)	50th	min–max	n	Mean (SD) or n (%)	50th	min–max	
Area home									
Urban residence		183 (91.5)				432 (86.7)			
Semi-urban		15 (7.5)				35 (7)			
Rural		2 (1)				31 (6.2)			
Current home									
Years of living		6.7 (7.6)	3	1–32		11.1 (9.7)	9	1–42	***
Owned		110 (55.0)				387 (77.7)			***
Rented		86 (43.0)				56 (11.2)			***
Other		9 (4.5)				72 (14.5)			
Type of home									
House		138 (69.0)				393 (78.9)			**
Flat		62 (31.0)				23 (4.6)			***
Shared						108 (21.7)			
Tenement						12 (2.4)			
Informal						43 (8.6)			
Other						125 (25.1)			
Fuel cooking									
Gas		200 (100)				492 (98.8)			
Wood		1 (0.5)				34 (6.8)			**
Coal						24 (4.8)			**
Electricity		1 (0.5)				6 (1.2)			
Other		4 (2.0)				2 (0.4)			*
Fuel heating									
Gas		195 (97.5)				73 (14.7)			***
Wood		5 (2.5)				22 (4.4)			
Electricity		11 (5.5)				242 (48.6)			***
Coal						27 (5.4)			
None						152 (30.5)			
Other		13 (6.5)				4 (0.8)			***
Drinking water, sources									
Running/community tap		163 (81.5)				491 (98.6)			***
Well, rain, river		1 (0.5)				1 (1.4)			
Other sources (bottled)		56 (28.0)				5 (1.0)			***
People in home	199	4.3 (1.6)	4	2–15	497	6.6 (3.5)	6	1–23	***
Partner, permanent job	187	177 (94.7)			338	325 (96.2)			
Partner, education	186				340				***
Primary education		19 (10.2)				144 (42.4)			
Secondary education		106 (57.0)				169 (49.7)			
Tertiary education		25 (13.4)				17 (5.0)			
University education		35 (18.8)				8 (2.4)			
No education		1 (0.5)				2 (0.6)			
Harvesting own food									
Growing own food	200	4 (2.0)			491	52 (10.6)			***
Go fishing (family)		67 (33.5)				139 (28.1)			
Eat their own fish		41 (61.2)				101 (71.6)			
Pollutants									
Lead use repairing home	200	53 (26.5)			497	231 (46.5)			**
do not know		57 (28.5)				28 (5.6)			
Pollution around home	200	18 (9.0)			494	277 (56.1)			***
Pesticide insect control home	195	9 (4.6)			497	398 (80.1)			***
Pesticide use garden	197	2 (1.0)			490	53 (10.8)			***
Control programme insects^b^	200				467	157 (33.6)			

^a^*, **, *** represent p-value <0.05, <0.010, <0.001, respectively; ^b^Changas, Dengue, Yellow Fever or Malaria Control Programme.

Gas was the primary home cooking fuel in both communities; while it dominated as the heating fuel in Salta, electricity did so in Ushuaia (see Table 3). Running/community tap water were the dominating sources of drinking water in both Salta (96%) and Ushuaia (81%); for the latter, bottled water was the secondary source. Growing/harvesting of food was seldom done (11% in Salta and 2% in Ushuaia) and the consumption of caught fish was relatively common (60–70%) in both places.

Almost half of the Salta women and very few from Ushuaia reported use of lead-containing materials during home repair. Environmental pollution sources in the vicinity of dwellings was more widespread in Salta (56%) than in in Ushuaia (9%) and the use of pesticides for insect control in homes was most common in Salta (80%), where a control programme for chagas, yellow fever or malaria existed.

**Figure 2.**
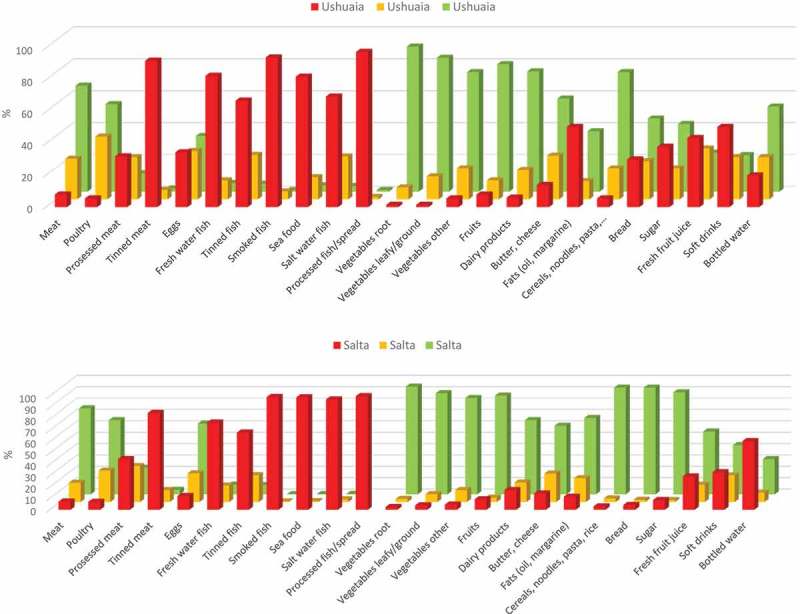
Maternal intake (%) of dietary items in (a) Ushia and (b) Salta. The intake frequencies depicted were significantly different between the two communities at p < 0.05 or better, with the exception of vegetables roots, fruits and butter/cheese (p ≥ 0.05) (see Supplementary Table S1).

Details about the food frequency intake during pregnancy are reported in Supplementary Table S1 and illustrated in Figure 2(a,b). There were almost no differences in frequency intake before (not shown) or during during pregnancy, nor between the communities for items like vegetables (root and leafy), butter and cheese and noodles, rice and pasta. Women in Salta reported a more frequent intake of meat, eggs, vegetables, cereals, fats and sugar. Fish intake was generally low for both sites, with women from Ushuaia eating slightly more, although there was somewhat higher intake of freshwater and tinned fish in Salta.

**Figure 3.**
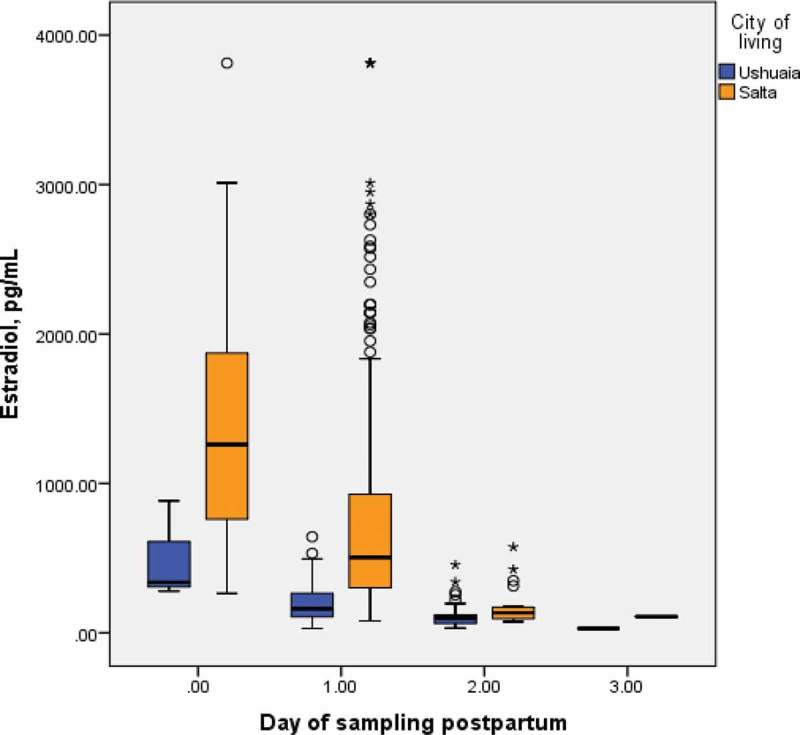
Distribution of maternal estradiol (pg/mL) by day of sampling post-partum.

Haematological data are presented in Table 4 and significant differences (p < 0.001) are evident for all. The clinical chemistry data for CRP, ferritin, total cholesterol, triglyceride and LDL were higher in Ushuaia, while those of estradiol were lower (p<0.001). The serum estradiol data depicted in Figure 3 indicate that significant changes occurred during the first days post-partum.

**Table 4.** Concentrations of maternal serum CRP, ferritin, lipids and estradiol sampled at a median of 1-day post partum.

	Ushuaia (n = 200)						Salta (n = 498)						p-value
	n	Mean	SD	Median	Min	Max	n	Mean	SD	Median	Min	Max	
C-reactive protein (CRP), mg/L	199	56	42	45	3	280	471	41	33	31	5	331	<0.001
Ferritin, ng/mL	199	36	32	26	6	245	464	18	16	13	2	181	<0.001
Cholesterol, total, mg/dL	199	225	65	219	88	602	471	210	45	203	119	394	<0.001
Triglycerides, mg/dL	199	220	76	207	72	488	471	183	68	173	53	496	<0.001
Phospholipids, mg/dl							471	208	45	201	118	357	
HDL Cholesterol, mg/dl							471	47	12	47	22	137	
LDL Cholesterol, mg/dl	196	134	53	131	33	386	471	127	35	128	12	297	<0.001
Estradiol, pg/mL^a^	199	181	124	148	29	883	470	731	672	499	74	3813	<0.001

^a^ The concentrations mU/mL of follicle-stimulating hormone (FSH) and luteinising hormone (LH) were mostly at around the detection limit.

## Discussion

Differences in socio-economic status and in regional development between the southern area (Ushuaia) and the poorer region of Salta are evident. The latter has more unemployment and a different social structure. Salta had a higher birth rate (16% being para four or more), longer breastfeeding periods and less use of vitamin and folic acids supplements, both before and during pregnancy. The single case of anaemia in Salta, compared to two in Ushuaia, seems inconsistent with the lower serum ferritin concentrations observed there. Perhaps this and the absence of reported cases of gestational diabetes and hypertensive disorders in Salta may well reflect under-reporting or under-diagnosis. In Ushuaia, 43% Caesarean deliveries were registered compared to only 6% in Salta and this reflects its globally established association with a higher socio-economic status [21]. The elevated CRP concentrations observed in Ushuaia presumably reflect the higher rate of Caesarean deliveries [22].

In a preliminary analysis of the EMSAR data [23], a limited set of organochlorine compounds (OCs) in blood serum indicated dependencies of the concentrations on BMI, parity and region of residence. It is generally recognised that lactation transfers OCs to a newly born baby [24]. There is also enough evidence that diet constitutes an important source of such environmental contaminants [9]. More detailed analyses of the full EMASAR study data set on concentrations, sources and pathways of both OCs and inorganic substances (including essential elements) will be presented in forthcoming papers. Regional differences in exposure to POPs and inorganic elements will be evaluated, respectively, by comparisons of the more historic *p,p′*-DDT/*p,p′*-DDE ratio and selected PCB isomer ratios in sera, as well as of pertinent toxic-to-essential element ratios in whole blood [25].

Although the personal and lifestyle information obtained and reported is somewhat limited, we feel that the described data set is adequate to examine in some detail the influence/modulation of maternal body burdens of PTS and foetal exposure in relation to maternal characteristics, parity, breast feeding history, living and environmental conditions/issues [26,27]. Potential relationships of PTS to the clinical chemistry parameters measured are anticipated, such as the association of OCs with circulating hormones such as estradiol [28,29] and the influence of reduced maternal lipid concentrations post-partum [30,31], as well as factors that can enhance the uptake of inorganic elements, such as low iron status [25]. Associations of the maternal body burdens of organochlorine and inorganic toxicants with birth weight, birth length and gestational age of the newborns will also be examined.

## Concluding remarks

The documented misuse of pesticides in agricultural production and food production is a real threat to the food security, especially for those of reproductive age and pregnant women, since the most vulnerable period in human life is during pregnancy and early childhood [18]. There is an urgent need for a comprehensive assessment of exposures in areas of the Southern Hemisphere. We conclude that our comprehensive data set and the planned publications of observed concentrations of inorganic and organic environmental contaminants in both mothers and their newborns, as well as their interpretation and the anticipated follow-up of the children, will contribute to this objective.

## Supplementary Material

Supplemental_Materials.docx
