# Supplementary material for: The Argentinian mother-and-child contaminant study: a cross-sectional study among delivering women in the cities of Ushuaia and Salta
Source: Int J Circumpolar Health. 2017 Aug 28;76(1):1364598. doi: 10.1080/22423982.2017.1364598 (PMC5645782; doi:10.1080/22423982.2017.1364598)
Supplement: Supplemental_Materials.docx [file ZICH_A_1364598_SM8885.docx]

**Supplemental Material**

# The Argentinian Mother-and-Child Contaminant Study: a cross-sectional study among delivering women in the cities of Ushuaia and Salta

^1^Inger Økland, ^2^Jon Øyvind Odland*, ^3^Silvinia Matiocevich, ^4^Marisa Viviana Alvarez, ^5^Torbjørn Aarsland, ^6^Evert Nieboer, ^2^Solrunn Hansen

^1^Department of Obstetrics and Gynecology, Stavanger University Hospital, Stavanger, Norway; ^2^Department of Community Medicine, Faculty of Health Sciences, UiT The Arctic University of Norway, Tromsø, Norway; ^3^Banco de Sangre, Clínica San Jorge, Ushuaia, Argentina; ^4^Hospital Público Materno Infantil, Salta, Argentina; ^5^Department of Research, Stavanger University Hospital, Stavanger, Norway; ^6^Department of Biochemistry and Biomedical Sciences, McMaster University, Hamilton ON, Canada

*jon.oyvind.odland@uit.no
